# Development of a dual energy CT based model to assess response to treatment in patients with high grade serous ovarian cancer: a pilot cohort study

**DOI:** 10.1186/s40644-023-00579-2

**Published:** 2023-06-15

**Authors:** Zena Alizzi, Andrew Gogbashian, Emmanouil Karteris, Marcia Hall

**Affiliations:** 1 Mount Vernon Cancer Centre, Rickmansworth Road, HA6 2RN Northwood Middx, England; 2 Brunel University London, Kingston Lane, UB3 8PH Uxbridge, England; 3 Paul Strickland Scanner Centre, Rickmansworth Road, HA6 2RN Northwood, Middlesex England

**Keywords:** Dual energy CT, Iodine concentration, High grade serous ovarian cancer (HGSOC), GCIG CA125, RECIST, Progression free survival (PFS)

## Abstract

**Background:**

In patients with cancer, the current gold standard for assessing response to treatment involves measuring cancer lesions on computed tomography (CT) imaging. The percentage change in size of specific lesions determines whether patients have had a complete/partial response or progressive disease, according to RECIST criteria. Dual Energy CT (DECT) permits additional measurements of iodine concentration, a surrogate marker of vascularity. Here we explore the role of changes in iodine concentration within cancer tissue on CT scans to assess its suitability for determining treatment response in patients with high grade serous ovarian cancer (HGSOC).

**Methods:**

Suitable RECIST measurable lesions were identified from the CT images of HGSOC patients, taken at 2 different time points (pre and post treatment). Changes in size and iodine concentration were measured for each lesion. PR/SD were classified as responders, PD was classified as non-responder. Radiological responses were correlated with clinical and CA125 outcomes.

**Results:**

62 patients had appropriate imaging for assessment. 22 were excluded as they only had one DECT scan. 32/40 patients assessed (113 lesions) had received treatment for relapsed HGSOC. RECIST and GCIG (Gynaecologic Cancer Inter Group) CA125 criteria / clinical assessment of response for patients was correlated with changes in iodine concentration, before and after treatment. The prediction of median progression free survival was significantly better associated with changes in iodine concentration (p = 0.0001) and GCIG Ca125 / clinical assessment (p = 0.0028) in comparison to RECIST criteria (p = 0.43).

**Conclusion:**

Changes in iodine concentration from dual energy CT imaging may be more suitable than RECIST in assessing response to treatment in patients with HGSOC.

**Trial Registration:**

CICATRIx IRAS number 198179, 14 Dec 2015, https://www.myresearchproject.org.uk/.

**Supplementary Information:**

The online version contains supplementary material available at 10.1186/s40644-023-00579-2.

## Introduction

Approximately 70% of high grade serous ovarian cancer (HGSOC) patients present with advanced (Stage III/IV) disease. HGSOC occurs most commonly in females between 75 and 79 years. Overall, 72% OC patients survive 1 year but only 43% are still alive at 5 years [1]. To optimise and improve survival rates for patients with HGSOC, radical primary surgery and effective relapse treatments are required. Maintenance therapies such as antiangiogenics and PARP inhibitors are also making significant contributions. However, determining the extent of abdominal disease, to identify those most suitable for primary surgery and to assess objective responses to relapse and especially novel therapies, in the context of clinical trials, remains a challenge. Many modalities of imaging have been studied, yet CT remains the gold standard because of its reliable reproducibility, widespread availability, cost efficiency and fast scanning times [2]. Conventional CT imaging is unable to detect small (< 5 mm) deposits which in HGSOC particularly, often results in reports containing ‘non-measurable’ findings such as ‘haziness, streaking, nodularity, thickening’ to describe disease covering the bowel serosa, mesentery or peritoneum, for example. These descriptions are subjective and yet are often the only imaging evidence of disease [3].

The currently accepted method for assessing a cancer patient’s response to treatment are the Response Evaluation Criteria in Solid Tumours (RECIST) [4]. These depend on accurate measurement of a specific mass. The more diffuse pattern of HGSOC especially in the peritoneal space precludes target lesion measurement. The ‘soft’ appearances, including ascites and pleural effusion, of HGSOC cannot be objectively measured and are considered ‘not RECIST assessable’. RECIST alone remains the most commonly used primary endpoint in clinical trials, as regulatory agencies remain fixated on anatomical changes in size of cancer lesions for registration trials [5, 6]. Given the difficulties with objective assessments of response in HGSOC patients a significant proportion of otherwise appropriate patients are excluded from trial entry. For this reason, definitions for response and progression in ovarian cancer trials utilising CA125 measurements (with and without RECIST 1.1 assessments) have been developed and agreed by the Gynaecological Cancer Intergroup (GCIG) [7]. Frustratingly, there is low concordance between CA125 and RECIST responses, and CA125 responses are often more reflective of clinical improvement [8]. Outside of clinical trials, a combination of symptomatic improvement, CA125 changes and imaging are used. Divergent outcomes are frequently encountered between the three parameters but treatment is rarely discontinued if a patient’s symptoms have improved and CA125 has fallen or stabilised. Controversially, RECIST alone remains the most commonly used primary endpoint in ovarian cancer clinical trials, as regulatory agencies remain fixated on anatomical changes in size of cancer lesions for registration trials [9, 10].

Conventional CT scans use single energy frequency (~ 120kVp) to establish the extent of disease [3]. Dual energy CT (DECT) allows the simultaneous collection of data from different photon spectra. The composition and density of different substances have discrete appearances, determined by the ability of individual chemical constitutions to absorb high and low energy frequencies (generally 80/140 kV) [11]. By changing photon energy level, information on the tissue composition can be elucidated [12]. Lower tube voltage (e.g. 80–100 kVp) results in improved iodine contrast enhancement because the mean photon energy approaches the iodine k-edge of 33 keV [13]. The main constituents of human soft tissues are hydrogen, carbon, oxygen and nitrogen. These have similar atomic numbers and are challenging to differentiate. The introduction of contrast such as iodine with substantially divergent absorption frequencies, denotes increased perfusion, strongly correlated with proangiogenic malignant tissue [14, 15]. Thus, measuring iodine concentration is a surrogate for vascularity, perfusion and permeability. Iodine maps, representing iodine concentrations, correlate with blood vessel distribution, including uptake in hypervascular cancer lesions [16, 17]. Angiogenesis has long been implicated in the development and progression of HGSOC and treatment with the VEGF inhibitor, bevacizumab has demonstrated efficacy in patients with HGSOC [18–20]. The aim of this study is to determine the value of DECT and measuring change in iodine concentrations from cancer lesions in HGSOC patients with respect to patient response to treatment. Responses according to RECIST and CA125 (GCIG criteria) will be correlated with iodine concentration changes.

## Materials and methods

### Patient population

**Fig. 1:**
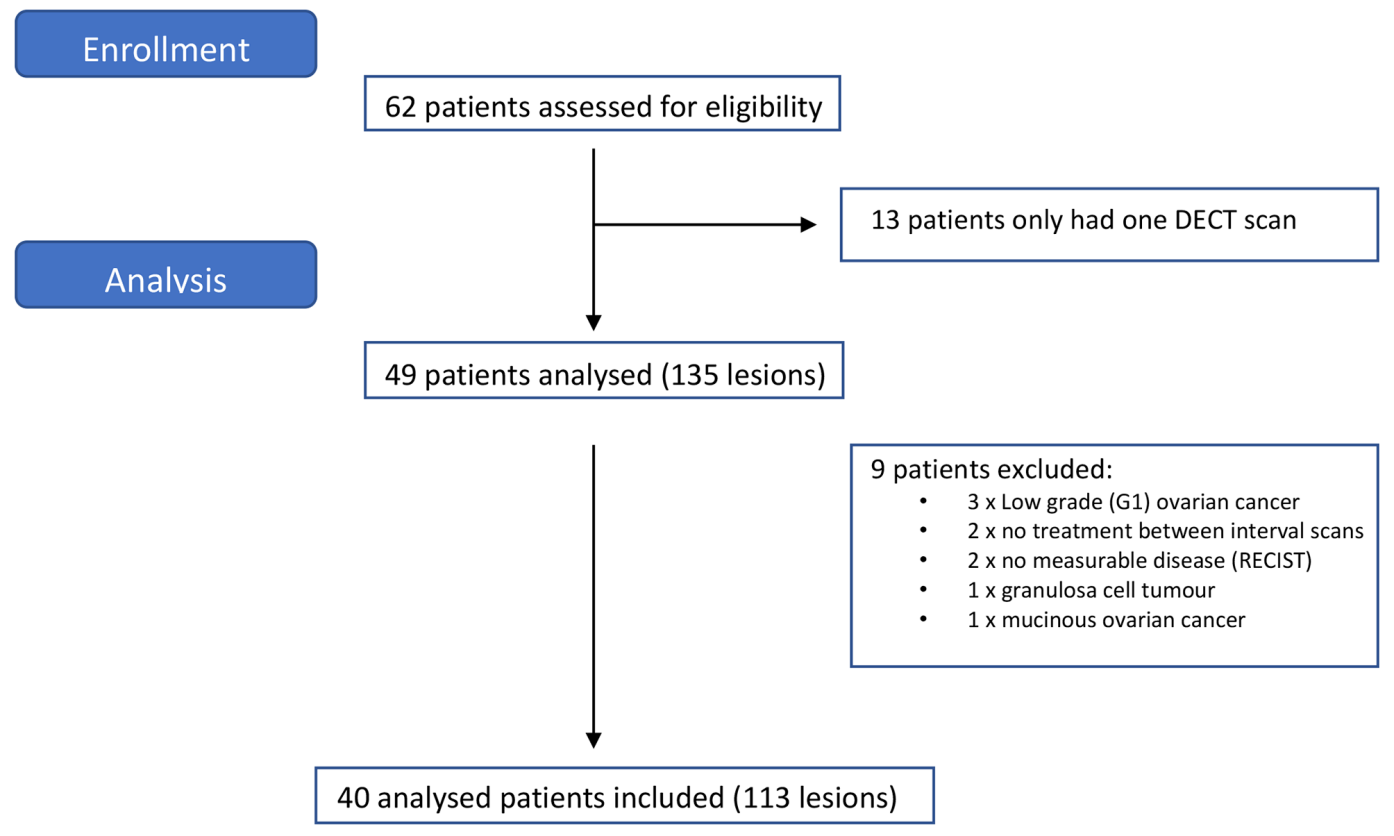
CONSORT diagram of patients included in analysis

In this cohort study, 62 patients diagnosed with HGSOC, who had DECT staging scans between 2016 and 2021, were identified. All patients were enrolled onto the CICATRIx trial (West Midlands–South Birmingham Ethics Committee Reference 16/WM/0196), at Mount Vernon Cancer Centre. This is an observational study looking at circulating cancer cells in peripheral blood in patients with HGSOC in collaboration with Brunel University. All enrolled patients also give consent to allow review of their imaging for research purposes. 40 patients were included and analysed, as 22 patients had only had one DECT imaging set and so were not evaluable (Fig. 1). Included patients all had imaging undertaken approximately 3–6 monthly: before, occasionally mid-point and at the end of a line of treatment. Concomitant CA125 values, clinical outcomes and RECIST assessments were obtained for the 40 eligible patients.

### Computed tomography and data analysis

Patients had CT examinations of the chest, abdomen and pelvis using a dual source CT scanner (Somatom Force, Siemens Healthcare, Forchheim, Germany). This system consists of two x-ray tubes mounted on one gantry. Patients were positioned supine on the table. Although some scanners using DECT can use slightly higher radiation doses than single energy scanners, with newer computer ‘dose-saving’ techniques built into them, many, such as ours, use similar or even reduced radiation doses compared to standard single energy CT. After intravenous injection of a non-ionic contrast agent (50-150mls Optiray 350 IV depending on patient weight, at a rate of 3ml/s), bolus tracking was started in the abdominal aorta at the level of the hemidiaphragms with a trigger of 100HU for the arterial phase of the chest. A 10s delay was applied to acquire the portovenous phase of the abdomen and pelvis from the dome of the liver to the iliac crests acquiring dual energy datasets with tube voltages between 90 kV and Sn150kV (tin filter). For both tubes, a dose modulation (Care DOSE 4D, Siemens Medical Solutions) was used. The reference mAs was set at 60 (90 kV), 46 (Sn150 kV) for the thorax and 190 (100 kV), 95 (sn150) for the abdomen/pelvis. A pitch of 0.55 was utilised. Conventional polychromatic images were reconstructed to contiguous axial slices (2 mm) with increment of 1.5 mm and measurable lesions identified. Lesions defined as measurable as per RECIST criteria were analysed for size, shape and location. These were mostly peritoneal but there were a few liver/splenic lesions. The ruler was used to measure the lesion size in the longest diameter of the solid part of the lesion- 113 such lesions were included in the analysis.

In addition, iodine-based, non-enhanced images were reconstructed using dedicated dual-energy processing software (Syngo Dual energy; Siemens Medical Solutions, Forhheim, Germany). An iodine map that codes the iodine distribution in individual CT voxels, representing the iodine concentration, was generated for evaluation, using this software. All lesions were measured for size, shape, density (HU) and iodine concentration (mg/ml), using the region of interest tool, encompassing the solid area of the lesion. Lesions were selected if they were measurable by RECIST criteria. Measurement of the iodine concentration was undertaken by placing the region of interest tool in the middle of the solid component of a lesion, with the aim of measuring approximately half of its surface area. We then standardised these lesions by normalising the iodine concentration to the aorta at the level of the diaphragm.

Image analysis and lesion choice was performed by a single reader (oncologist who chose and measured lesions previously identified by trained radiologists for reporting, under the close supervision of a radiologist with more than 10 years experience, who confirmed findings). Inter-reader agreement was not performed for this initial observational study.

### Patient-based evaluation of response

Treatment responses were assessed as per RECIST 1.1 criteria [4]: i.e. the percentage change in size was calculated using the sum of the longest dimension (SLD) for the measurable lesions and calculating the percentage change from baseline. Response is defined as follows: CR (complete disappearance of all lesions), PR (a 30% decrease in the SLD from baseline), PD (a 20% increase in the SLD from baseline) and SD (neither PR nor PD) [4].

Changes in CA125 levels in patients with HGSOC are used routinely to determine response to treatment. The strict criteria-defined GCIG CA125 changes are used in more formal clinical trial settings, often as secondary endpoints [7]. CA125 values obtained at the time of scans, or as close to the scan date as possible, were documented and CA125 GCIG criteria applied to determine CA125 response / progression: response is defined as a 50% reduction in CA125 levels from a pre-treatment sample (which must be elevated to at least 2 x upper limit of normal (ULN) prior to therapy) and maintained for at least 28 days – i.e. the 50% reduction in value is still seen in samples at least 28 days later. Patients with CA125 levels less than twice the ULN are not evaluable by GCIG CA125 criteria. Progression using CA125 is defined as an increase in CA125 to at least twice the nadir value or ULN, measured on 2 occasions at least a week apart [7].

Determination of response, stable disease and progression were documented for all three methods of evaluation in every patient. Changes in individual patient iodine concentration measurements were compared to the same patients’ RECIST and GCIG CA125 responses. Progression free survival (PFS) was determined by calculating the time between the date of treatment initiation and dates of progression as per RECIST, GCIG CA125 and iodine concentration measurements.

### Statistical analyses

Patients were categorised into two groups- responders and non-responders. Responders are defined as patients fulfilling RECIST, GCIG CA125 or newly designated iodine concentration (mg/ml) (or DECT) criteria for complete/partial response or stable disease. Non-responders were classified as those with progressive disease according to either RECIST, GCIG CA125 or iodine concentration. The values of mean percentage changes in tumour size (RECIST) and iodine concentration (DECT) were calculated for the responder and non-responder groups. The changes were then correlated with response calculated by GCIG CA125 criteria. To evaluate the ability for RECIST, GCIG CA125 and the new DECT criteria in predicting PFS (in months) were compared between the groups, with responders and non-responders categorised by each response criterion, by using a log-rank test. Statistical analyses were carried out using GraphPad Prism software. Kaplan-Meier survival curves were used to determine whether iodine concentration is more sensitive at predicting response in comparison to RECIST criteria.

## Results

The baseline characteristics of the 40 patients analysed are summarized in Suppl. Table 1. 37 patients had HGSOC, 3 were high grade endometrioid ovarian cancer and all were stage III/IV at diagnosis. 80% had relapsed HGSOC disease at the time of their pre-treatment DECT scan. 65% received chemotherapy for their OC and 35% were receiving maintenance treatments including PARP inhibitors, hormone or immune-therapy.

### Determination of a DECT response criteria

**Fig. 2:**
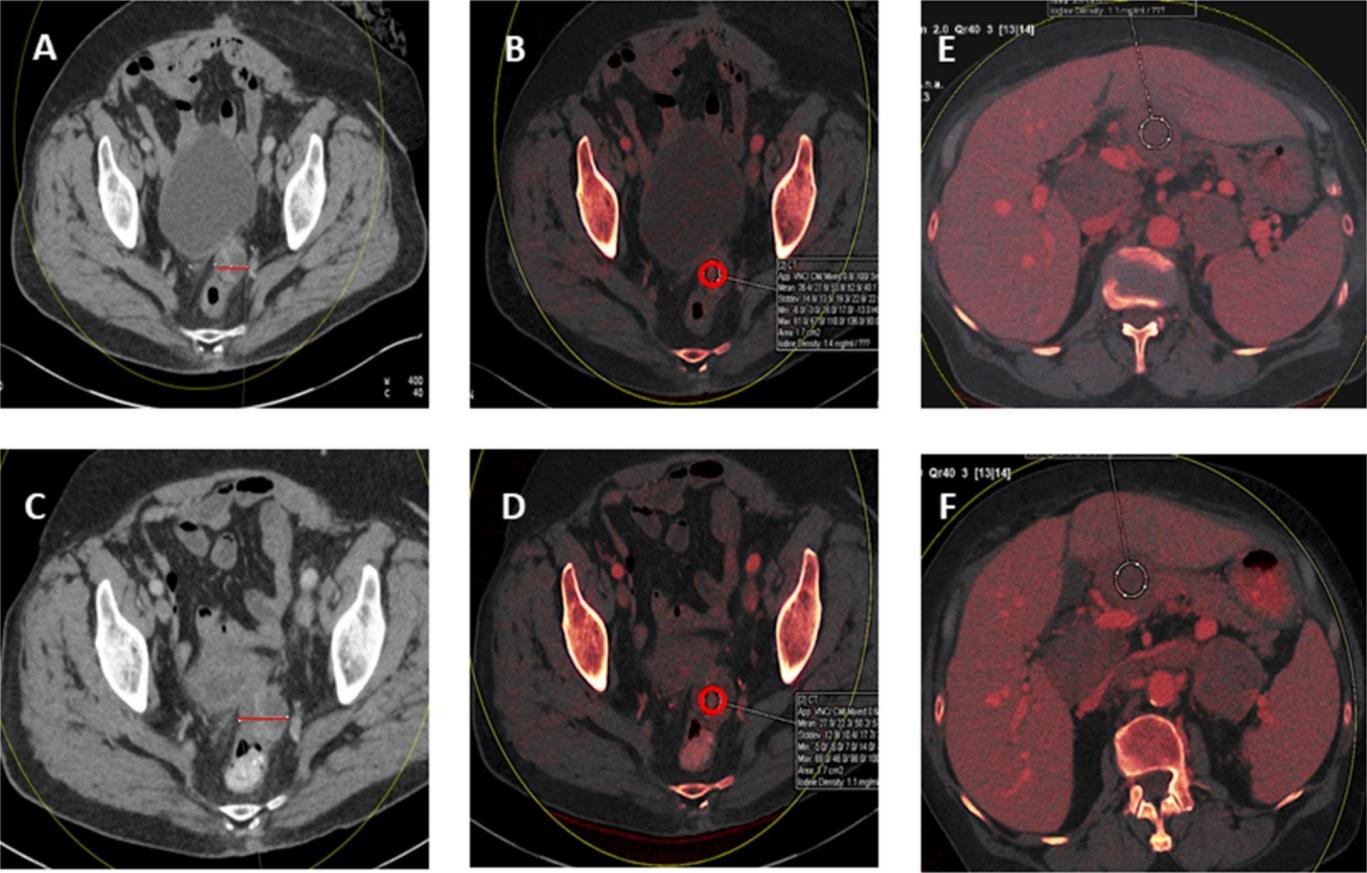
DECT axial imaging with 120kv reconstruction **(A/C)** and iodine maps **(B/D)** obtained at 90kv and 150kv –soft tissue mass, posterior to bladder, pre- treatment: **(A)** Lesion measures 2.98 cm, **(B)** iodine concentration − 1.4 mg/mL and post-treatment: **(C)** Lesion measures 3.5 cm and **(D)** iodine concentration − 1.1 mg/mL. Iodine maps **(E/F)** obtained at 150 kV- peritoneal deposit adjacent to quadrate/left lobe of liver, pre-treatment **(E)** iodine concentration 1.1 mg/mL, mid-treatment **(F)** iodine concentration 0.9 mg/mL

Figure 2 details an example of the methods of evaluation of a lesion from DECT images for a patient, using standard anatomical RECIST v 1.1 and an iodine map with freehand ROI before (A, B) and after (C, D) treatment respectively. Counter-intuitively, the measured lesion here increased in size when assessed by RECIST criteria, yet quantitatively, iodine concentration reduced, suggesting the reduction in vascularity here is more representative of response than lesion size – which here maybe influenced by an increase in volume of encysted fluid. Images E/F also demonstrate very little change, and possibly an increase in size, in the peritoneal deposit if assessed by RECIST, yet the iodine concentration reduces. This patient was responding to her chemotherapy clinically and by GCIG CA125 criteria despite no obvious RECIST evaluable change.

Percentage changes in size of the target lesion(s) were evaluated for each patient and response designated according to RECIST criteria [4]. CA125 results, before and after the same treatments period, were obtained for each patient and responses designated according to GCIG CA125 criteria. Non-responders included those with new or enlarging lesions on follow up imaging, as per RECIST 1.1, and/or those with increases in CA125 values to twice the ULN or nadir, as per GCIG CA125 criteria. Those who experienced a therapy change or died within 6 months of their follow up examination were also classified as non-responders. Non-responders demonstrated an increase in iodine concentrations. Evaluations of differing percentage values including 10%, 15% and 20% were applied to this training cohort. A cut off value of 15% of iodine concentration was selected, as this best reflected the clinical outcomes in these patients. However, it was noted that patients with *any* decrease in iodine concentration demonstrated clinical improvement so these patients were also considered “responders” and a reduction of ≥ 15% was considered to amount to a partial response.

**Fig. 3:**
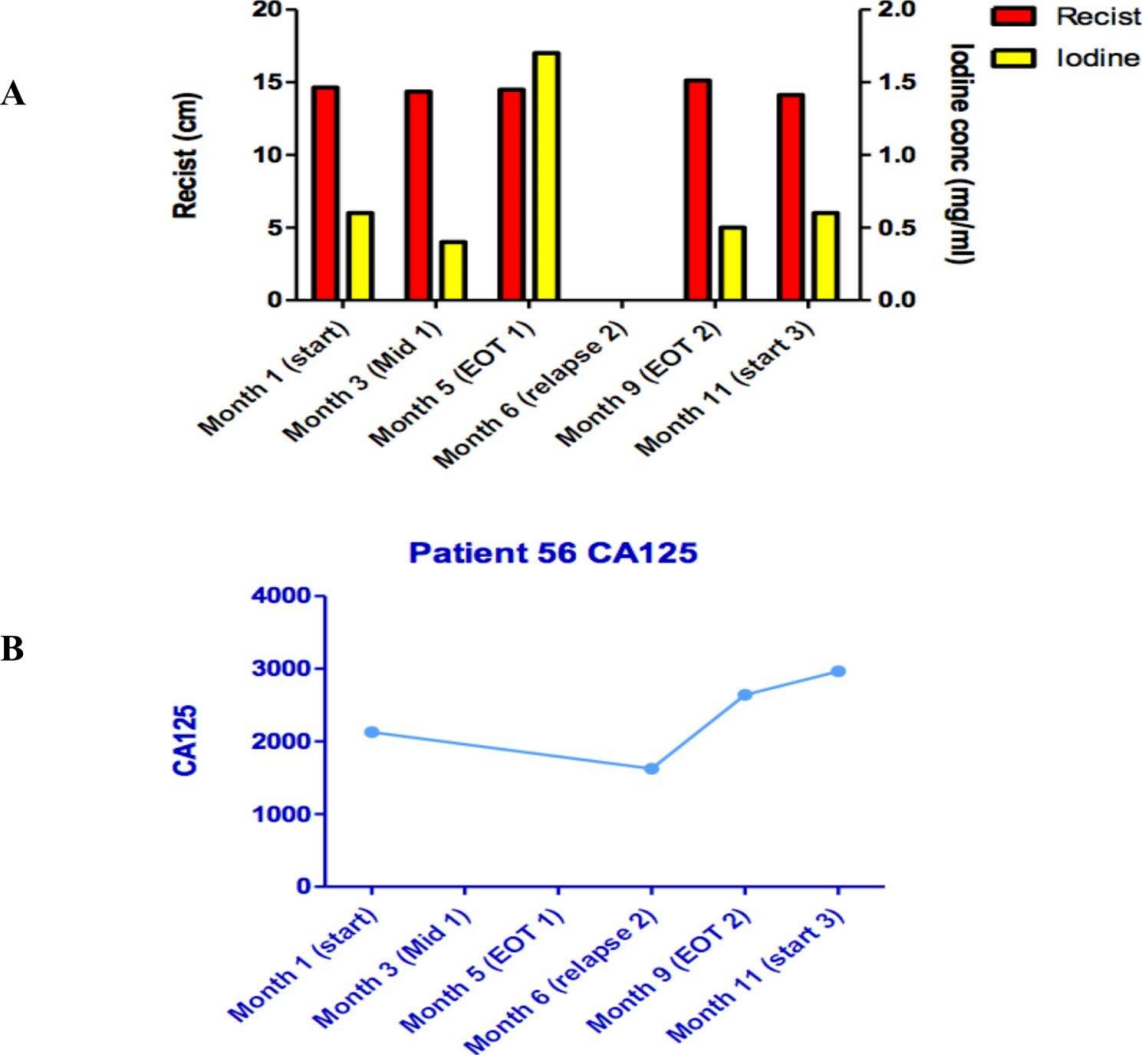
Fig. **3A** changes in size and iodine concentration of cancer lesion from one patient over ~ 12 months during which this patient received three different lines of treatment. RECIST and CA125 GCIG (**3B**) evaluations suggested stable disease throughout but the iodine concentration changes aligned more closely with the patient’s clinical condition

An example of RECIST evaluation (size measurements) in comparison with iodine concentration changes for the same lesions in a single patient who underwent three lines of therapy over a period of ~ 12 months is shown in Fig. 3. RECIST measurements remained stable over the three treatment periods, as did the CA125 values. However, there was a modest fall in iodine concentration midway through the first treatment (month 3), in line with clinical improvement in the patient’s symptoms. At the end of the first treatment a significant rise in iodine concentration (month 5) presaged the requirement for further chemotherapy the following month for symptomatic relapse.

### Value of different response criteria

Amongst the 40 patients, 60% had stable disease by RECIST criteria. only 16/40 patients (40%), demonstrated sufficient size changes in target lesions to be assigned as partial response or progressive disease as per RECIST. (Suppl. Table 2).

Of 11 patients who had progressive disease as assessed by a ≥ 15% increase in iodine concentration, only 4 also had > 20% increase in size, consistent with progression by RECIST criteria. Of the remaining patients with DECT progression, 5 had less than 20% increase and 2 patients had a decrease in size. There was better concordance for the group assessed as progressive disease by iodine concentration with GCIG CA125 response criteria: 9/11 (82%) patients fulfilled GCIG CA125 criteria for progressive disease whilst 2 patients had stable disease by GCIG Ca125 criteria (Suppl.Table 2).

Amongst patients fulfilling the newly designated DECT criteria for partial response, i.e. ≥ 15% reduction in iodine concentration, 5/8(63%) patients also achieved the required reductions in size for RECIST partial response criteria. Two of the remaining 3 patients demonstrated reductions in size of their target lesion but < 30%. The final patient’s target lesion increased in size (< 20%). Again, there was improved concordance with the GCIG CA125 response criteria, 6/8 (75%) patients demonstrated reductions in CA125 amounting to CR/PR, the final 2 had stable disease by GCIG CA125 (Suppl. Table 2).

### Survival analysis

More important than response to both clinicians and patients is the length of time that patients live following treatment before their cancer symptoms return, requiring further therapy. Thus, PFS is a critical endpoint in many clinical trials. Arguably stable disease, for a few months is a better outcome than a partial response lasting a few weeks. 32 of the 40 patients were receiving treatment for relapsed OC, the remaining 8 had imaging before and after first line chemotherapy. We have therefore explored the PFS for the 32 relapse OC patients, correlating this with each of the three methods of treatment response evaluation and including patients with stable disease as responders.

**Fig. 4:**
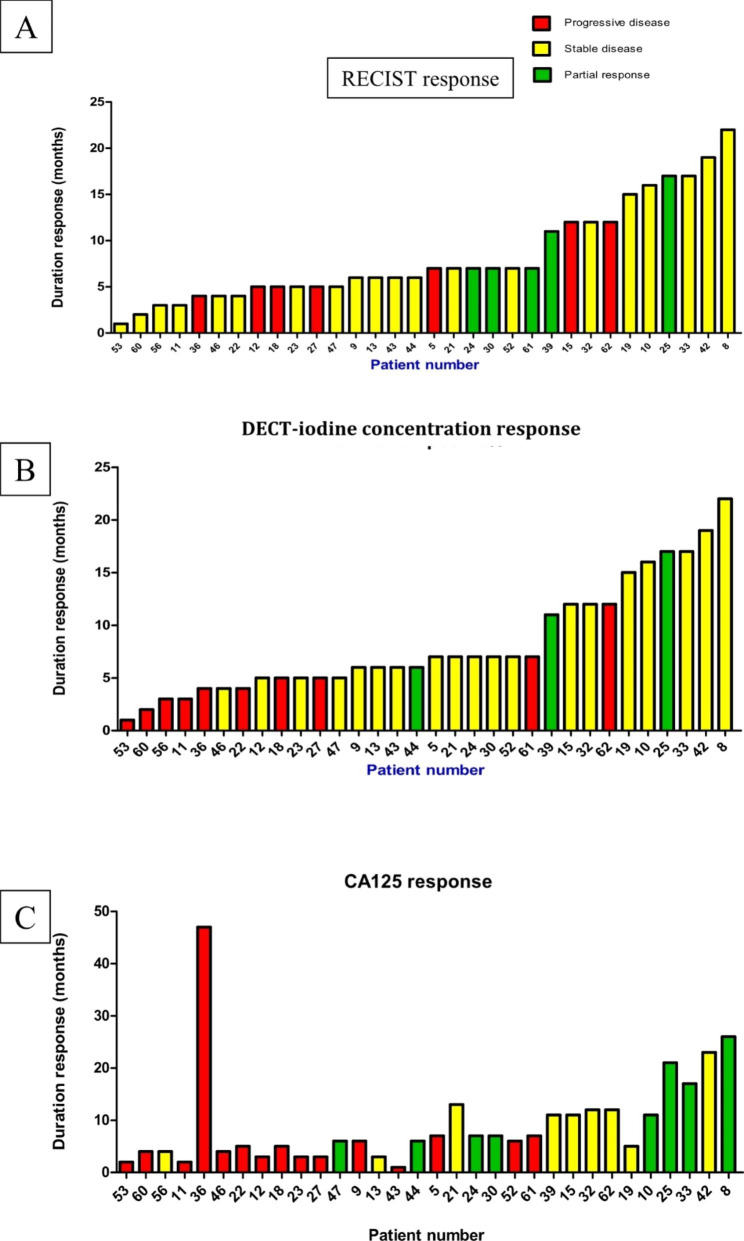
Percentage change as per RECIST/DECT-iodine concentration/CA125 criteria aligned with duration of response (months) for each relapsed patient. **A)** RECIST response, **B)** DECT- iodine concentration response, where 15% increase/reduction in concentration is designated a ‘response’ and **C)** GCIG CA125 response

**Fig. 5:**
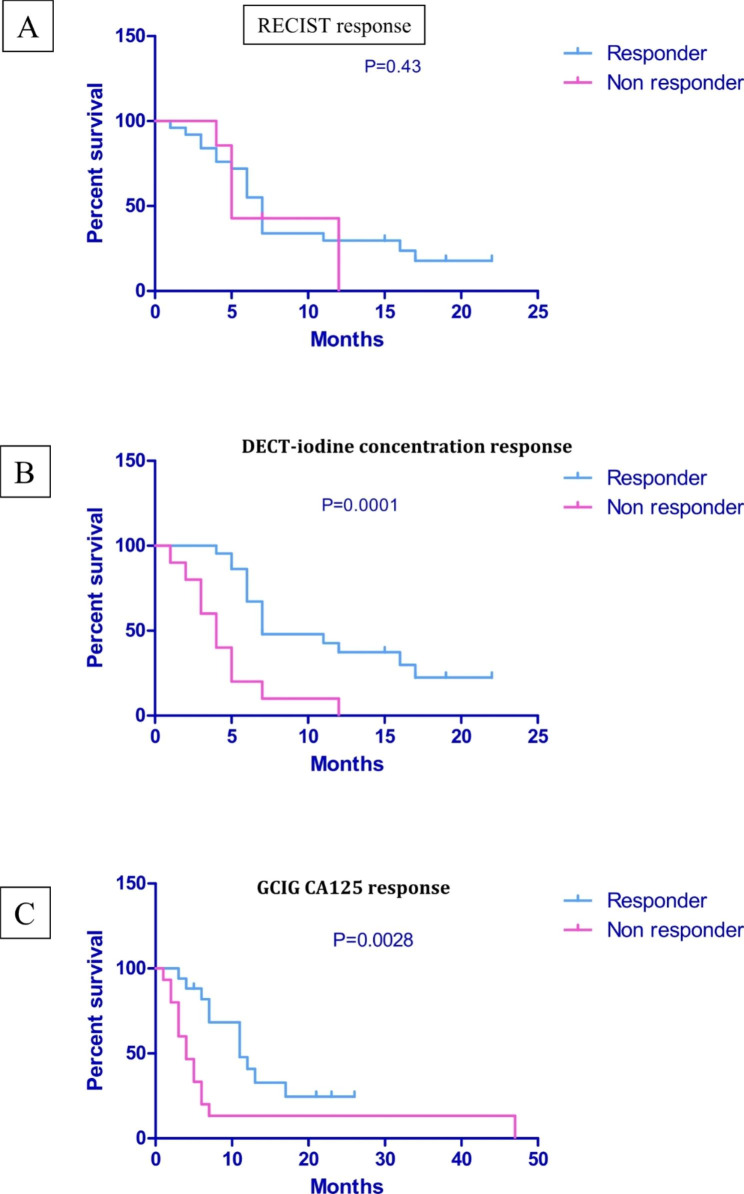
Progression Free Survival (PFS) according to response in n = 32 relapse patients. **A:** RECIST v1.1 median PFS 7 months (responder, n = 25) versus 5 months (non responders, n = 7) p = 0.43 HR 0.7 95% CI 0.24 to 2.05. **B:** DECT (iodine concentration) median PFS 7 months (responders, n = 22) versus 4 months (non responders, n = 10) p =  0.0001, HR 0.1 95% CI 0.03 to 0.33. **C:** GCIG CA125 Median PFS 11 months (responders, n = 17) versus 4 months (non responders, n = 15) p =  0.0028, HR 0.23 95% CI 0.09 to 0.6

78% of the 32 relapse patients had stable disease or a partial response according to RECIST, 53% had a response according to GCIG CA125 criteria and 69% had a reduction in DECT-iodine concentration (Fig. 4). The PFS for these patients according to RECIST (A) and DECT-iodine concentration (B) is demonstrated in Figs. 4 and 5. Median survival according to RECIST for responders was 7 months versus 5 months for non-responders (p = 0.43, HR 0.7 95% CI 0.24 to 2.05). Median survival according to any reduction in DECT-iodine concentration was 7 months for responders and 4 months for non-responders (p = 0.0001, HR 0.1 95% CI 0.03 to 0.33). (C) Median survival according GCIG CA125 was 11 months for responders and 4 months for non-responders (p = 0.0028 h 0.23 95% CI 0.09 to 0.6).

## Discussion

There are now many initiatives underway to explore the textural aspects of cross-sectional imaging using computational approaches, which more readily reflect tumour heterogeneity [21]. Subtle surface alterations in larger masses may reflect significant changes in smaller volume disease; it is recognised that in some instances imaging changes in tumour / stromal density and micro vessel density contrast enhancement correlate better with clinical response [11]. Choi et al. explored gastrointestinal stromal tumours (GIST) and developed criteria involving changes in tumour density (using Hounsfield Units), together with smaller changes in size (10%) [6]. These Choi criteria are more successful at identifying patients with GIST (rare mesenchymal tumours arising from the GI tract) who are responding. Although not perfect, these criteria are now accepted practice in this setting.

DECT is increasingly installed as standard in many new CT scanners potentially offering easy access to information about tissue composition in patients. This strategy also allows reduction of artefacts such as calcification and metallic prostheses [22]. Numerous studies have demonstrated a clear link between micro vessel density and iodine concentration confirming this as a marker of vascularity. Invasive cancers typically have higher levels of iodine concentration in comparison to normal tissue due to their rapid growth and active angiogenesis [23]. Iodine maps can characterise vascularised lesions such as haemangioma and differentiate these from hepatocellular carcinoma [24]. Using DECT in 19 patients also in patients with GIST, Meyer et al., demonstrated that iodine concentration changes predicted duration of response better than RECIST or Choi criteria [25]. Similarly, in head and neck and gastric cancers, iodine concentration changes utilising DECT determines tumour boundaries better for surgical / radiation purposes and can be used to determine response [26–28]. In patients with oesophageal cancer changes in iodine maps before and after chemoradiation are a significantly better method of response assessment than RECIST [29]. Finally, as we have described here, Kawamoto et al. reported small reductions in iodine concentration in association with clinical improvements in patients receiving chemotherapy for pancreatic cancer compared with progression seen in all patients where iodine concentration increased [30]. This supported our attribution of patients with any decrease in iodine concentration as “responders”.

Amongst our cohort of EOC patients, patients with disease progression (PD) by DECT-iodine concentration criteria, had a significantly shorter duration of response (< 6 months). The superiority of DECT iodine concentration to detect PD is advantageous when compared to RECIST as in clinical practice, where the best evidence of clinical benefit is an absence of progression [31]. Such DECT-iodine concentration changes are also more reflective of changes in CA125 and clinical symptoms. By contrast, RECIST (Fig. 4A) identifies many patients who required further treatment within 6 months, as having stable disease. Although this work did not compare DECT with alternate cross sectional imaging techniques, it is recognised that PET-CT fails to recognise the true extent of peritoneal spread, especially the mesentery and bowel serosa, and to date there is only limited data on the impact of the newer MRI techniques on clinical decision making [32].

There are several limitations to this study. This is a single centre, retrospective study with a small sample size, which was unselected with respect to treatment received. Further, a single reader (oncologist) collected the measurements, albeit closely supervised by an experienced radiologist. Larger validation cohorts from multiple centres with dual reporting are necessary to confirm these findings. Sample bias is possible- i.e. that by choosing patients with RECIST assessable disease, we have identified a population also susceptible to changes in iodine concentration. This seems unlikely given that there is no correlation with median PFS and RECIST assessment where there is a significant correlation with iodine concentration. It should also be noted that GCIG CA125 criteria have only been validated for use with relapse chemotherapy or first-line disease progression. Here we apply them to some ROC patients receiving hormonal and/or molecular / biological agents. Similarly, the use of a mixed cohort of patients receiving different treatments could have affected these results. However, this pilot data supports future work where the impact of treatments can be explored. It is important to note that none of the patients studied here were receiving antiangiogenics as these are not funded in the UK for relapse OC. Finally, the evaluation of avascular cystic masses remains challenging; here, only solid areas were chosen for iodine concentration analysis.

## Conclusion

In conclusion, changes in iodine concentration appears to be an alternative method of measuring treatment outcomes for patients with HGSOC. Such changes correlate more closely with these HGSOC patients clinical and GCIG CA125 criteria responses than with the RECIST designations of response. Changes in iodine concentration may be better than RECIST in predicting duration of response in such patients, especially those for whom the treatment is not effective (i.e. determining progressive disease more reliably). More accurate objective response methodology than RECIST is urgently required for patients with HGSOC. The wide availability of DECT built into standard CT scanners should be a stimulus to undertake this. These data can serve as a training set for the prognostic value of response with regard to PFS to be carried out in an independent group of patients.

## Electronic supplementary material

Below is the link to the electronic supplementary material.

**Supplementary Table 1**: Baseline characteristics

**Supplementary Table 2**: Distribution of response classification by RECIST, DECT- iodine concentration and CA125

## Data Availability

Datasets and material analysed are available on reasonable request from the corresponding author.

## Abbreviations

CA125: Cancer Antigen 125
CR: Complete Response
CT: Computed Tomography
DECT: Dual Energy Computed Tomography
GCIG: Gynaecological Cancer Inter-Group
GIST: Gastro-Intestinal Stromal Tumour
HGSOC: High Grade Serous Ovarian Cancer
HU: Hounsfield Unit
IV: IntraVenous
KeV: Kilo electron Volt
kV: Kilovolt
kVp: Kilovoltage peak
mls: Millilitres
mm: Millimetres
OC: Ovarian Cancer
PARP: Poly (ADP Ribose) Polymerase
PD: Progressive Disease
PFS: Progression Free Survival
PR: Partial Response
RECIST: Response Evaluation Criteria in Solid Tumours
s: second
SD: Stable Disease
SLD: Sum of Longest Diameters
Sn: Tin (filter applied for beam hardening)
ULN: Upper Limit Normal
VEGF: Vascular Endothelial Growth Factor
