# Supplementary material for: Development of a dual energy CT based model to assess response to treatment in patients with high grade serous ovarian cancer: a pilot cohort study
Source: Cancer Imaging. 2023 Jun 15;23:62. doi: 10.1186/s40644-023-00579-2 (PMC10268407; doi:10.1186/s40644-023-00579-2)
Supplement: Supplementary file 1 — Supplementary Table 1: Baseline characteristics [file 40644_2023_579_MOESM1_ESM.docx]

|  | N=40 (%) |
| --- | --- |
| **Mean age (years)** | 61 |
| **Stage** |  |
| FIGO III | 31 (77.5) |
| FIGO IV | 9 (22.5) |
| **Treatment stage** |  |
| Neoadjuvant/adjuvant | 8 (20) |
| Relapse | 32 (80) |
| **Treatment** |  |
| Chemotherapy | 26 (65) |
| Hormones | 5 (12.5) |
| Immunotherapy | 3 (7.5) |
| Niraparib | 6 (15) |

**Suppl Table 1**: Baseline characteristics
