# Supplementary material for: Development of a dual energy CT based model to assess response to treatment in patients with high grade serous ovarian cancer: a pilot cohort study
Source: Cancer Imaging. 2023 Jun 15;23:62. doi: 10.1186/s40644-023-00579-2 (PMC10268407; doi:10.1186/s40644-023-00579-2)
Supplement: Supplementary file 2 — Supplementary Table 2: Distribution of response classification by RECIST, DECT- iodine concentration and CA125 [file 40644_2023_579_MOESM2_ESM.docx]

| N=40 | **RECIST** | **DECT (iodine concentration)** | **CA125** |
| --- | --- | --- | --- |
| Complete response | 0 | 0 | 9 |
| Partial response | 9 | 8 | 4 |
| Stable disease | 24 | 21 | 12 |
| Progressive disease | 7 | 11 | 15 |

**Suppl. Table 2:** Distribution of response classification by RECIST, DECT- iodine concentration and CA125
